# NADPH oxidase 4 is required for the generation of macrophage migration inhibitory factor and host defense against *Toxoplasma gondii* infection

**DOI:** 10.1038/s41598-017-06610-4

**Published:** 2017-07-25

**Authors:** Ji Hye Kim, Jina Lee, Su-Jin Bae, Yeeun Kim, Byung-Joon Park, Jae-Won Choi, Jaeyul Kwon, Guang-Ho Cha, Heon Jong Yoo, Eun-Kyeong Jo, Yun Soo Bae, Young-Ha Lee, Jae-Min Yuk

**Affiliations:** 1 Department of Infection Biology, College of Medicine, Chungnam National University, Daejeon, South Korea; 2 Department of Medical Science, College of Medicine, Chungnam National University, Daejeon, South Korea; 3 Department of Medical Education, College of Medicine, Chungnam National University, Daejeon, South Korea; 4 Department of Obstetrics and Gynecology, College of Medicine, Chungnam National University, Daejeon, South Korea; 5 Department of Microbiology, College of Medicine, Chungnam National University, Daejeon, South Korea; 6 Department of Life Science, Ewha Womans University, Seoul, South Korea

## Abstract

Nicotinamide adenine dinucleotide phosphate (NADPH) oxidases (Nox) are an important family of catalytic enzymes that generate reactive oxygen species (ROS), which mediate the regulation of diverse cellular functions. Although phagocyte Nox2/gp91phox is closely associated with the activation of host innate immune responses, the roles of Nox family protein during *Toxoplasma gondii* (*T*. *gondii*) infection have not been fully investigated. Here, we found that *T*. *gondii*-mediated ROS production was required for the upregulation of macrophage migration inhibitory factor (MIF) mRNA and protein levels via activation of mitogen-activated protein kinase and nuclear factor-κB signaling in macrophages. Interestingly, MIF knockdown led to a significant increase in the survival of intracellular *T*. *gondii* in bone marrow-derived macrophages (BMDMs). Moreover, Nox4 deficiency, but not Nox2/gp91phox and the cytosolic subunit p47phox, resulted in enhanced survival of the intracellular *T*. *gondii* RH strain and impaired expression of *T*. *gondii*-mediated MIF in BMDMs. Additionally, *Nox4*-deficient mice showed increased susceptibility to virulent RH strain infection and increased cyst burden in brain tissues and low levels of MIF expression following infection with the avirulent ME49 strain. Collectively, our findings indicate that Nox4-mediated ROS generation plays a central role in MIF production and resistance to *T*. *gondii* infection.

## Introduction

*Toxoplasma gondii* (*T*. *gondii*) is an obligate protozoan parasite that infects a broad range of warm-blooded animals, including avian and mammalian species^1^. It is believed to infect approximately one-third of the global population; however, most infected and otherwise-healthy people remain asymptomatic due to control via diverse immune defense systems^2^. Conversely, *T*. *gondii* exposure in immunocompromised and congenitally infected individuals can result in several neurological conditions and ocular toxoplasmosis, which are associated with considerable morbidity and mortality^2^. Earlier studies suggested that various cytokines related to Th1-mediated immune responses were required for host survival and persistence^3, 4^. Despite cooperation among immune defense mechanisms to eliminate *T*. *gondii*, it can actively invade and replicate in non-phagocytic and phagocytic cells through the actin/myosin-based motility system and the formation of parasitophorous vacuoles (PVs), respectively^5, 6^.

Reactive oxygen species (ROS) are generated by many cell types in the human body and are involved in a wide range of biological functions, such as cell survival, differentiation, apoptosis, and energy balance^7^. Moreover, increasing evidence suggests that ROS play essential roles not only as direct killing effectors of microbes but also as secondary messengers for intracellular signaling pathways related to the host immune response against invading microorganisms^8, 9^. Upon pathogen recognition by innate immune receptors, the nicotinamide adenine dinucleotide phosphate (NADPH) oxidase protein complex is activated by an association between cytoplasmic subunits (p47phox, p67phox, p40phox, Rac in phagocytes) and the membrane-bound heterodimer flavocytochrome b558 (Nox2/gp91phox and p22phox), which then generates superoxide and derivative forms by electron transfer, from NADPH to O_2_^10^. In addition to the catalytic subunit in phagocytic cells, Nox2/gp91phox, six other homologs, Nox1, Nox3, Nox4, Nox5, Duox1, and Duox2, have been identified in other tissues and cells. Genetic defects in the Nox2 system are closely associated with the pathogenesis of chronic granulomatous disease (CGD), which leads to recurrent infection by various microbes and abnormal formation of tissue granulomas because of impaired innate immune defenses^11^. Earlier studies showed that the Nox2 protein and its components were important for host protection against mycobacterial and Salmonella infections^12, 13^. Park *et al*. reported that Toll-like receptor 4 (TLR4)-induced ROS generation and activation of NF-κB signaling were mediated through the direct association of TLR4 with Nox4^14^. However, the precise roles of NADPH oxidase family proteins in the innate immune response against *T*. *gondii* infection have not been investigated fully.

Macrophage migration inhibitory factor (MIF) is a cytokine also known previously as glycosylation-inhibiting factor, L-dopachrome isomerase, and phenylpyruvate tautomerase; it is evolutionarily conserved in diverse species, including human, mouse, and cat^15^. MIF was first identified as a lymphokine produced by activated T lymphocytes to inhibit the random migration of macrophages. However, more recent studies have demonstrated that MIF is expressed constitutively in various cells, such as monocytes, macrophages, and endothelial cells, and has multiple activities in inflammatory responses and host anti-microbial immunity^16–18^. MIF is also involved in the pathogenesis and host immune responses to infection with various intracellular parasitic protozoa, including *Plasmodium* ^19^, *Leishmania* ^20, 21^, *Trypanosoma* ^22^, and *Toxoplasma* ^23, 24^.

In the present study, we examined the precise role of Nox family proteins in innate immune control against *Toxoplasma* infection using primary macrophages and an *in vivo* murine model. We found that Nox4-deficient C57BL/6 mice were more susceptible to a virulent RH strain and showed an increased parasite burden in brain tissue with an avirulent ME49 strain. We also found that the lack of Nox4, but not Nox2 or p47phox, in macrophages resulted in increased intracellular survival of the RH strain and decreased levels of MIF protein and mRNA. To further investigate the anti-toxoplasma effects of MIF in macrophages, intracellular survival of *T*. *gondii* and mRNA expression of *sag1* in *Mif*-knockdown BMDMs were assessed by confocal microscope and real-time PCR analysis, respectively. Additionally, *T*. *gondii* stimulation resulted in rapid activation of NF-κB and mitogen-activating protein kinases (MAPKs), and the generation of intracellular ROS in macrophages, which is important for *T*. *gondii*-mediated MIF expression. Our findings indicate that Nox4-derived ROS are an important mediator of MIF generation for anti-parasitic defence.

## Material and Methods

### Cell preparation

Bone marrow-derived macrophages (BMDMs) were differentiated for 5–7 days in medium containing macrophage colony-stimulating factor, as described previously^25^. The culture medium consisted of Dulbecco’s modified Eagle’s medium (DMEM, Life Technologies, Grand Island, NY, USA) supplemented with 10% heat-inactivated fetal bovine serum (FBS, Gibco BRL, Grand Island, NY, USA), 1 mM sodium pyruvate, 50 U/mL penicillin, 50 μg/mL streptomycin, and 5 × 10^−5^ M β-mercaptoethanol. The mouse macrophage cell line RAW264.7 and the human retinal pigment epithelial cell lines ARPE-19 were purchased from the American Type Culture Collection (ATCC, Manassas, VA, USA) and grown in DMEM supplemented with 10% FBS or with nutrient mixture F-12, 10% FBS and 1% Antibiotic-Antimycotic (Anti-Anti, Gibco BRL). ARPE-19 cells were passaged by 0.25% Trypsin-EDTA (Life Technologies, Carlsbad, CA, USA) every 2–3 days.

### Parasite preparation

Tachyzoites of *T*. *gondii* RH strain were maintained in ARPE-19 cells at 37 °C, 5% CO2 and biweekly passaged in DMEM with 10% FBS, nutrient mixture F-12, antibiotics. Tachyzoites expressing green fluorescent protein (GFP-RH strain) were kindly provided by Dr. Yoshifumi Nishikawa (Obihiro University of Agriculture and Veterinary Medicine, Japan). Bradyzoites of *T*. *gondii* ME49 strain were harvested from the brains of C57BL/6 or BALB/C mice that had been inoculated with 50 cysts and maintained every 3 weeks.

### Experimental *in vivo* murine models

Wild-type (WT) C57BL/6 mice were purchased from Koatech (Korea). Mice with a targeted deletion in the *Nox2*, *p47phox*, or *Nox4* gene (homozygous mice and their homozygous littermates) were generated as described previously^12, 26^. For *in vivo* experiments, mice were intraperitoneally (i.p.) infected with either 200 tachyzoites of the RH strain or 40 cysts of ME49 strain. Cysts in brain homogenates were obtained at 20 days after ME49 strain infection. The mRNA expression for *T*. *gondii*-specific gene *Sag1* (forward: 5′-CGGTGACAGTACAAGCCAGA-3′; reverse: 5′-CTTCCGCAGACAACTTGACA-3′) in brain homogenates was performed using specific primers.

### Ethics Statement

Animal experimental procedures were approved by the Institutional Animal Care and Use Committee (IACUC) at Chungnam National University (CNU-00706) and conformed to National Institutes of Health guidelines. The animals were fed standard rodent food and water ad libitum, and housed (maximum of 5 per cage) in sawdust-lined cages in an air-conditioned environment with 12-hour light/dark cycles. Animal husbandry was provided by the staff of the IACUC under the guidance of supervisors who are certified Animal Technologists, and by the staff of the Animal Core Facility. Veterinary care was provided by IACUC faculty members and veterinary residents located on the Chungnam National University School of Medicine.

### Reagents, antibody and DNA

For *in vitro* experiments, N-acetylcysteine (NAC), diphenyleneiodonium (DPI), BAY11-7082 (BAY), caffeic acid phenethyl ester (CAPE) were from Calbiochem (San Diego, CA, USA). 4,5-dihydroxy-1,3-benzene disulfonic acid disodium salt (Tiron) and dimethyl sulfoxide (DMSO; added to the cultures at 0.05% (v/v) as a solvent control) were from Sigma-Aldrich.

Specific antibodies against phospho-ERK1/2 (9101), phospho-p38 (9211), phospho-SAPK/JNK (9251), and phospho-IKKα/β (2681) were purchased from Cell Signaling. Specific antibodies against IκBα (sc-371) and NF-κB p65 (sc-372) were purchased from Santa Cruz Biotechnology. Specific antibodies against MIF (ab175189) and β-tubulin (ab6046) were purchased from Abcam. All other reagents were purchased from Sigma-Aldrich, unless otherwise indicated. The reporter plasmids pNF-kB-Luc and pAP1-Luc were kindly provided by Dr. Gang Min Hur (Chungnam National University).

### Lentiviral shRNA generation and transduction of primary cells

For silencing of target genes used in all experiments in primary cells, pLKO.1-based lentiviral *Mif* (clone ID TRCN0000067343 to TRCN0000067347) shRNA constructs were purchased from Open Biosystems. Lentiviruses were produced by transient transfection using packaging plasmids (pMDLg/pRRE, pRSV-Rev, and pMD2.VSV-G, purchased from Addgene) after Lipofectamine 2000-mediated transient transfection into HEK293T cells, as described previously^25^. Virus-containing medium samples were collected at 72 h post-transfection, filtered, and concentrated by ultracentrifugation. Titration of the lentiviral vectors was determined using HEK293T cells and the lentiviral vectors were transduced into the cells, as described previously^25^.

### RNA extraction, real-time quantitative PCR, semi-quantitative RT-PCR, Western blot analysis, and enzyme-linked immunosorbent assays (ELISAs)

RNA extraction, real-time quantitative PCR, and semi-quantitative RT-PCR were performed as described previously^25^. The sequences of the primers used were as follows: mMIF (forward: 5′-CTCTCCGAGCTCACCCAGCAG-3′; reverse: 5′-CGCGTTCATGTCGTAATAGTT-3′), mTNFα (forward: 5′-AGCACAGAAAGCATGATCCG-3′; reverse: 5′-CTGATGAGAGGGAGGCCATT-3), mIL-1β (forward: 5′-CTCCATGAGCTTTGTACAAGG-3′; reverse: 5′-TGCTGATGTACCAGTTGGGG-3′), mIL-12p40 (forward: 5′-GACCATCACTGTCAAAGAGTT-3′; reverse: 5′-AGGAAAGTCTTGTTTTTGAAA-3′), IFNGR1 (forward: 5′-TGTTACCTAAGTCCTTGCTC-3′; reverse: 5′-TCTTCCTGTTCTGCTGCTTC-3′), CCR5 (forward: 5′-TGCACAAAGAGACTTGAGGCA-3′; reverse: 5′-AGTGGTTCTTCCCTGTTGGCA-3′), TLR4 (forward: 5′-TTCAGAACTTCAGTGGCTGGA-3′; reverse: 5′-CTGGATAGGGTTTCCTGTCAGT), and mβ-actin (forward: 5′-TCATGAAGTG TGACGTTGACATCCGT-3′; reverse: 5′-CCTAGAAGCATTTGCGGTGCACGATG-3′).

For Western blot analysis, cell lysates were collected and lysed in PRO-PREP (iNtRON BIOTECHNOLOGY, Korea) containing additional set of phosphatase inhibitors. Protein concentration was determined using a BCA assay kit. Proteins (30 μg/each conditions) were immediately heated for 5 min at 100 °C. Each sample was subjected to SDS-PAGE on gel containing 12% (w/v) acrylamide under reducing conditions. Separated proteins were transferred to PVDF membranes (Millipore Corp., Billerica, MA, USA), and then the membranes were blocked with 5% skim milk. Membranes were developed using chemiluminescence assay kit (ECL; Millipore Corp., Billerica, MA, USA) and subsequently analyzed using Chemiluminescence Imaging System (UVitec Cambridge, MA, USA). Data were analyzed using Alliance Mini HD6 (UVitec Cambridge, MA, USA).

In the sandwich ELISA, serum and cell culture supernatants were analyzed using DuoSet antibody pairs (BD Pharmingen) for the detection of mouse MIF (DY1978). TNF and IL-12p40 in serum were measured using a Mouse BD OptEIA Set ELISA Kit (BD Biosciences, TNF: 555268, IL-12p40: 555165).

### Immunofluorescence microscopy of NF-kB p65 translocation

Translocation of NF-kB p65 into the nucleus was detected using immunofluorescence staining as previously described^27^. Briefly, cells were prepared on sterilized glass coverslips (BD Bio-sciences, Bedford, MA, USA) in triplicate and cells then were fixed in 4% paraformaldehyde in PBS for 10 min, permeabilized with 0.25% Triton X-100 in PBS for 10 min, and incubated with primary antibody (NF-κB p65; sc-372) for 2 h at room temperature. Cells were washed to remove excess primary antibody, and incubated with the appropriate fluorescently labeled secondary antibodies (anti-rabbit AlexaFluro 488) for 2 h at room temperature. Nuclei were stained by incubated with DAPI (Sigma) for 3 min. After mounting, fluorescence images were acquired using a confocal microscope (LSM 710; Zeiss).

### Measurement of ROS production

Level of intracellular ROS was determined by dihydroethidium (DHE, Calbiochem) or 2′,7′-dichlorodihydroflurescein (DCFDA, Invitrogen)^28^. Briefly, cells were incubated with 2 μM dihydroethidium (DHE, Calbiochem) for 15 min or 20 μM DCFDA for 30 min at 37 °C and then analyzed by a confocal microscope (LSM 710; Zeiss), a fluorescence microscope (Olympus BX-51, Tokyo, Japan), or a FACS Calibur flow cytometer (Becton-Dickinson, San Josè, CA, USA).

### Luciferase reporter assays

NF-kB and AP-1 luciferase reporter assay was performed as described previously^27^. Briefly, Raw 264.7 cells were transfected with plasmid containing NF-kB and AP-1 Luciferase (Genetransfer Vector Core; Iowa City, IA, USA) for 36 h, and the infected with *T*. *gondii* for indicated time periods. Infected cells were washed three times in PBS, and cell extracts were prepared by adding 100 μl of 1×Passive Reporter Lysis Buffer (Promega, Madison, WI, USA) Luciferase activity was measured using the Luciferase Assay System (Promega, Madison, WI, USA), according to the manufacturer’s instructions.

### Analysis of intracellular *T*. *gondii* Proliferation

BMDMs were seeded in 24-well plates with 22 mm glass coverslips and cells then were infected with GFP-RH strain by indicated time periods or moi. After indicated time, the coverslips were washed out with warmed PBS and fixed with 4% formaldehyde for 10 minutes at room temperature. Texas Red®-X phalloidin (Life Technologies Corporation, CA, USA) and 4′6-Diamidino-2-phenylindole (DAPI, Sigma) were used to stain cytosol and nucleus, respectively. Cover slides were mounted on the glass with Fluoromount-G (SouthernBiotech, Birmingham, USA) and analyzed by confocal microscopy (LSM 710; Zeiss). 10 sections were randomly selected and total number of infected cell or the number of parasite per vacuole was counted.

### Statistical analyses

All data were analyzed by Student’s *t*-test with Bonferroni adjustment or ANOVA for multiple comparisons and are presented as the means ± SD. Statistical comparisons were carried out using GraphPad Prism software (GraphPad Software, Inc. La Jolla, CA, USA) and the Tukey’s Multiple Comparison Test was used to determine one-way analysis of variance (ANOVA) procedures. Differences were considered significant at *p* < 0.05.

## Results

### Intracellular ROS-dependent MIF expression plays an essential roles in macrophages-mediated host defenses against *T*. *gondii* infection

**Figure 1:**
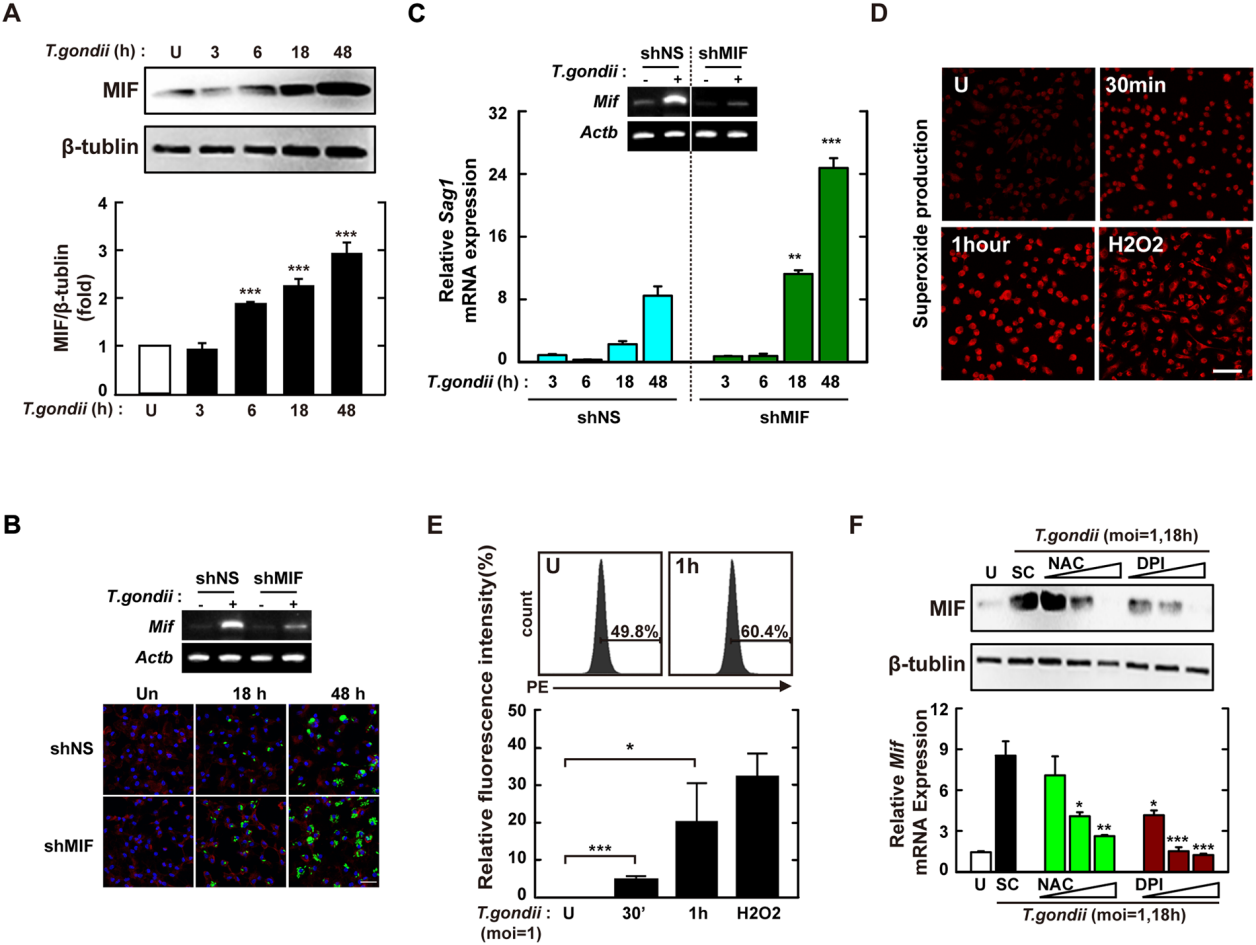
MIF expression via intracellular ROS generation is required for the inhibition of intracellular proliferation in *T*. *gondii*-infected macrophages. (**A**) BMDMs were infected with *T*.*gondii* RH strain (moi = 1) for the indicated time periods and then cell lysate was collected. Immunoblot analysis was performed for protein expression of MIF or β-tubulin. *Upper panel*, Representative gel image. *Lower panel*, Densitometry. (**B**,**C**) BMDM were transduced with lentiviruses expressing shNS or shMIF at a multiplicity of infection (MOI) of 5 for 48 h with polybrene (8 μg/mL) and then infected with GFP-RH strain (for B) or *T*. *gondii* RH strain (for C) for the indicated time periods. The mRNA expression for *Mif* and *Actb* was determined using semiquantitative RT-PCR (**B**) Cells were fixed and stained with Texas Red®-X phalloidin for labeling F-actin (red) for cytosolic fraction, and DAPI (blue) for nuclei and then analyzed for the number of GFP-RH strain using confocal microscopy (bottom) (**C**) Quantitative real-time PCR analysis were assessed to determine *sag1* mRNA expression in whole-cell lysates. (**D**,**E**) BMDMs were infected with *T*. *gondii* RH strain (moi = 1) for indicated times and then stained with DHE (2 μM) for 15 min. Intracellular ROS generation was measured using confocal microscopy (for D) and flow cytometery (for E). Scale bar = 50 μm. H_2_O_2_ (1 mM, 30 min) was used for positive control. (**F**) Immunoblot (top) or qPCR (bottom) analysis of MIF expression in BMDMs after *T*. *gondii* RH strain infection (moi = 1, 18 hr) in the presence or absence of general antioxidant (NAC; 1, 2, or 5 mM) or Nox inhibitor (DPI; 1, 5, or 10 μM). Data are representative of three independent experiments and are presented as means ± SD. *P < 0.05, **P < 0.01, ***P < 0.001, two-tailed Student’s t-test.

Because MIF participates in host resistance against various intracellular protozoa infections^15^, we assessed the expression pattern of MIF in response to *T*. *gondii* in primary macrophages. The results showed that *T*. *gondii*-mediated protein (Fig. 1A) and mRNA (Fig. S1A) MIF levels were induced initially, from 6 h after infection, and increased continually until 48 h in BMDMs. Additionally, *T*. *gondii*-infected BMDMs showed enhanced MIF protein (Fig. S1B) and mRNA (Fig. S1C) levels, in a concentration-dependent manner. We next investigated whether MIF contributes to host defence against *T*. *gondii* infection in BMDMs. BMDMs were transduced with various lentiviral short hairpin RNAs (shRNAs) against *Mif* (shMIF), and the efficiency of lentiviral transduction was assessed by semi-quantitative reverse transcription (RT)-PCR (Fig. S2, top). The *sag1* mRNA level was significantly increased in BMDMs transduced with shMIF clone 4 and 5 constructs (Fig. S2, bottom). In addition, intracellular survival and proliferation of GFP-tagged *T*. *gondii* (Fig. 1B) and the *sag1* mRNA level (Fig. 1C) were markedly higher in BMDMs transduced with shMIF than in those transduced with lentivirus expressing nonspecific shRNA (shNS).

We next examined whether intracellular ROS generation was required for *T*. *gondii*-mediated MIF activation. The generation of ROS in BMDMs was determined by measuring oxidized DCFDA (for hydrogen peroxide; Fig. S3) or dihydroethidium (DHE, for superoxide anion; Fig 1D and E) using flow cytometry and a laser-based confocal microscope, respectively. Exposure of BMDMs to *T*. *gondii* resulted in rapid generation of intracellular ROS, within 10–30 min, and peak activation at 1 h after infection (Fig. 1D, E and S3). Pretreatment with N-acetyl-cysteine (NAC, the general anti-oxidant) or diphenylene iodonium (DPI, irreversible inhibitor of flavoenzymes including NADPH oxidase) significantly attenuated *T*. *gondii*-mediated ROS generation (data not shown) and MIF activation (Fig. 1F). These results indicated that *T*. *gondii*-induced MIF expression was mediated by intracellular ROS generation.

### The c-Jun N-terminal kinases (JNK) and p38 MAPK pathway is required for MIF expression via ROS-mediated activation of transcription factor activator protein 1 (AP-1) in response to *T*. *gondii*

**Figure 2:**
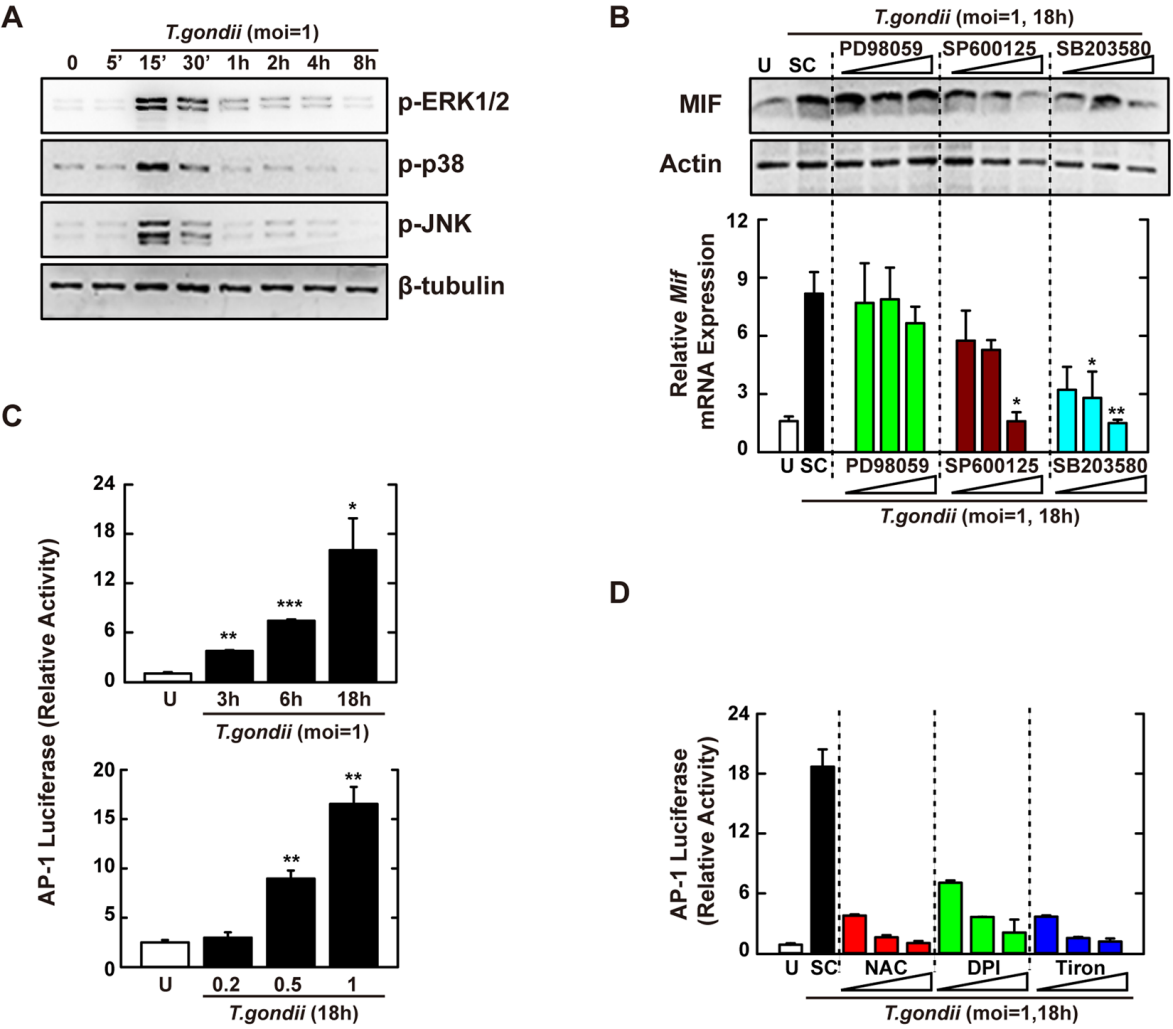
*T*. *gondii*-induced MIF expression is mediated through the activation of JNK and p38 MAPK pathway, followed by the AP-1 transcriptional activation. (**A**) BMDMs were infected with *T*. *gondii* RH strain (moi = 1) for the indicated time periods. Cells were harvested and subjected to western blotting analysis for phosphorylated ERK, p38, or JNK. β-tubulin served as a loading control. (**B**) BMDMs were pretreated with PD98059 (5, 10, 20 μM), SP600125 (5, 20, 30 μM) or SB203580 (1, 5, 10 μM) for 45 min, followed by infection with *T*. *gondii* RH strain (moi = 1) for 18 h. Immunoblot (top) or qPCR (bottom) analysis was performed to determine protein and mRNA expression MIF, respectively. (**C**) Raw 264.7 cells were transfected with plasmids carrying AP-1 luciferase reporter constructs before *T*. *gondii* RH strain (moi = 1, top; indicated moi, bottom) for various time periods (top) or 18 h. Luciferase assays were performed based on normalization to the β-galactosidase activity. (**D**) Effects on AP-1 transcriptional activity in the presence or absence of general antioxidant (NAC; 1, 2, or 5 mM), Nox inhibitor (DPI; 1, 5, or 10 μM) or superoxide scavenger (Tiron; 5, 10, or 20 mM). The experimental conditions were as outlined in Fig. 2C. Data are representative of three independent experiments and are presented as means ± SD. *P < 0.05, **P < 0.01, ***P < 0.001, two-tailed Student’s t-test.

MAPKs are known to be essential upstream kinases that activate the transcription factor AP-1^29^. Moreover, several putative DNA-binding sites, such as those for AP-1, specificity protein (Sp)1, and cyclic adenosine monophosphate (cAMP) response element-binding protein (CREB), are located within the promoter region of the *MIF* gene^30^. However, the molecular mechanism(s) involved in *T*. *gondii*-mediated upregulation of MIF expression have not been determined. To examine whether MAPK signaling was responsible for induction of the *MIF* gene, we first analyzed the activation of p38, extracellular signal–regulated kinases (ERK) 1/2, and JNK in response to *T*. *gondii* infection. Murine BMDMs infected with *T*. *gondii* showed strong phosphorylation of all three MAPK subfamilies at 30 min (Fig. 2A). Additionally, the *T*. *gondii*-mediated upregulation of the MIF protein was decreased by specific inhibitors of p38 MAPK (SB203580) and JNK (SP600125), but not ERK (PD98059; Fig. 2B, top). A similar effect was also observed in mRNA expression from the *Mif* gene, using real-time polymerase chain reaction (PCR) (Fig. 2B, bottom).

To assess the role of ROS in the activation of MAPK-dependent AP-1 signaling, Raw 264.7 macrophages were transfected with a luciferase reporter vector containing AP-1 response elements and then infected with *T*. *gondii* (multiplicity of infection, MOI = 1) for the time periods indicated (Fig. 2C, top) or with various MOI of *T*. *gondii* for 18 h (Fig. 2C, bottom). Macrophages infected with *T*. *gondii* showed enhanced transcriptional activity of AP-1 in a time- and MOI-dependent manner (Fig. 2C), which was concentration-dependently decreased in the presence of the general anti-oxidants NAC and tiron and the NADPH oxidase inhibitor DPI (Fig. 2D). These findings indicated that ROS-mediated activation of transcription factor AP-1 was essential for the induction of MIF via p38 MAPK and JNK, but not ERK, signaling pathways in macrophages.

### NF-κB signaling is important for *T*. *gondii*-induced MIF expression in macrophages

**Figure 3:**
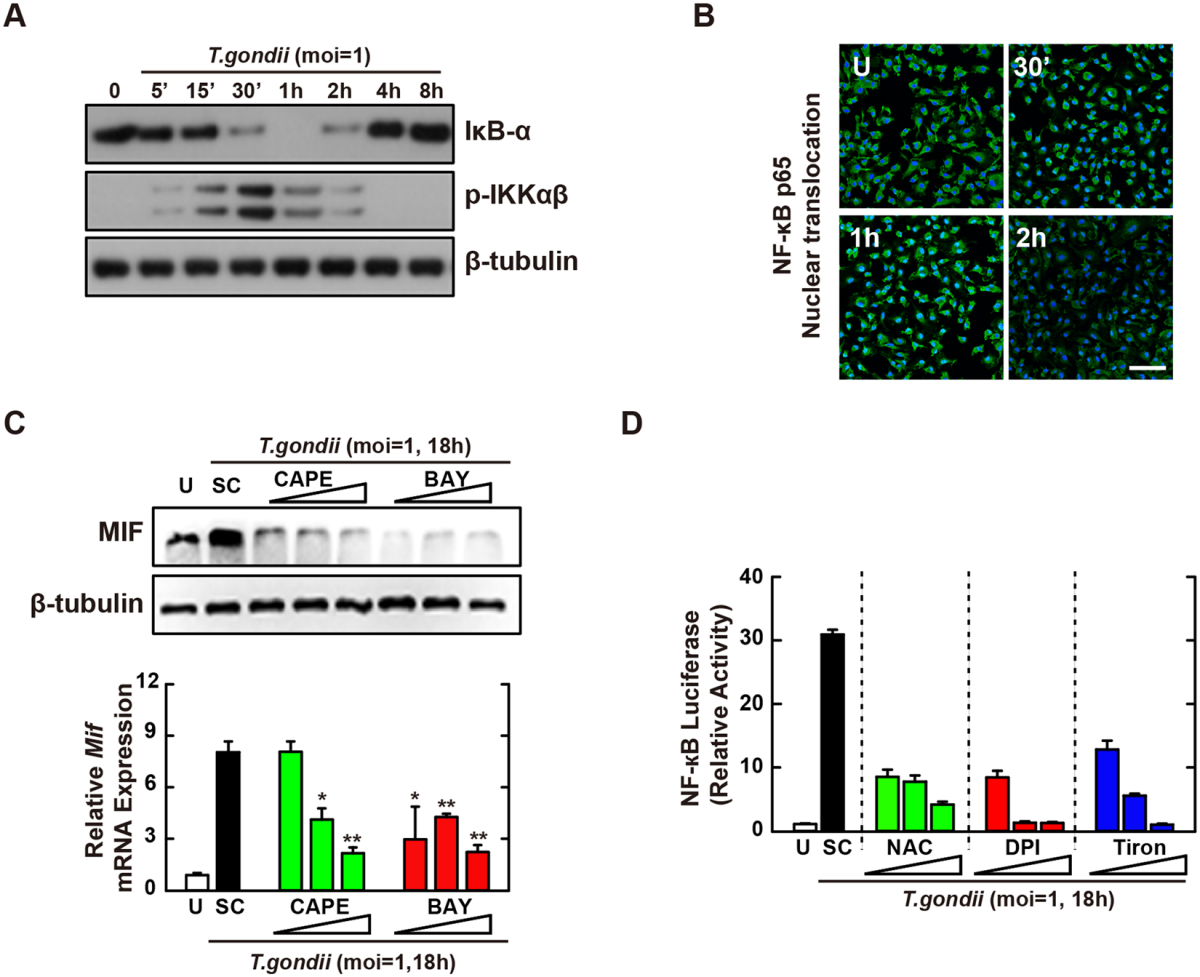
NF-κB signaling plays an essential role in *T*. *gondii*-induced MIF expression by BMDMs. (**A**,**B**) BMDMs were infected with *T*. *gondii* RH strain (moi = 1) for the indicated time periods. (**A**) Immunoblot analysis was performed to determine protein expression of total IκB-α and phosphorylated IKKα/β. β-tubulin served as a loading control. (**B**) Immunofluorescence analyses of NF-κB p65 nuclear translocation. Cells were fixed and stained with anti-NF-κB p65 (green); nuclei were stained with DNA-intercalating dye DAPI (blue). Scale bar = 50 μm. (**C**) BMDMs were pretreated with CAPE (1, 5 or 10 µM, 2 h) and BAY (0.1, 1, 3 μM, 45 min) and then infected with *T*. *gondii* RH strain (moi = 1) for 18 h. Immunoblot (top) or qPCR (bottom) analysis was performed to determine protein and mRNA expression MIF, respectively. (**D**) Raw 264.7 cells were transfected with plasmids carrying NF-κB luciferase reporter constructs. Cells were pretreated with general antioxidant (NAC; 1, 2, or 5 mM), Nox inhibitor (DPI; 1, 5, or 10 μM) or superoxide scavenger (Tiron; 5, 10, or 20 mM) and then infected with *T*. *gondii* RH strain (moi = 1) for 18 h. Luciferase activity were measured and normalized to β-galactosidase activity. Data are representative of three independent experiments and are presented as means ± SD. *P < 0.05, **P < 0.01, ***P < 0.001, two-tailed Student’s t-test.

Because the activation of NF-κB was closely related with MIF production^31, 32^, we next assessed whether NF-κB signaling was required for the upregulation of MIF expression in *T*. *gondii*-infected macrophages. *T*. *gondii* infection resulted in strong phosphorylation of IκB kinase (IKK) α/β within 15–30 min, following rapid degradation of IκBα (Fig. 3A). Moreover, NF-κB nuclear translocation was initiated at 10 min and then occurred markedly at 30–60 min after *T*. *gondii* infection in BMDMs (Fig. 3B). We next examined *T*. *gondii*-induced MIF expression in the presence of the NF-κB-specific inhibitors caffeic acid phenethyl ester (CAPE) and Bay 11-7085 (Bay). As shown in Fig. 3C, pretreatment with CAPE or Bay in *T*. *gondii*-infected BMDMs effectively attenuated the upregulation of MIF protein (Fig. 3C, top) and mRNA (Fig. 3C, bottom) levels in a concentration-dependent manner.

Because the promoter region of the *MIF* gene contains DNA-binding sites for transcription factor NF-κB as well as AP-1^30^, we performed a luciferase assay to evaluate the transcriptional activity of NF-κB following *T*. *gondii* infection. For this, Raw 264.7 cells were transfected with a luciferase reporter plasmid containing a response element for NF-κB. *T*. *gondii* infection strongly increased NF-κB transcriptional activities, in a time- and MOI-dependent manner (Fig. S4A and B). However, pretreatment with ROS inhibitors NAC, DPI, or Tiron resulted in marked reductions in NF-κB reporter gene activity in *T*. *gondii*-infected macrophages (Fig. 3D). Taken together, these results demonstrated that *T*. *gondii*-induced MIF expression in macrophages required the activation of NF-κB signaling via intracellular ROS generation.

### NADPH oxidase catalytic subunit Nox2/gp91phox is not required for MIF activation to control *T*. *gondii* infection

**Figure 4:**
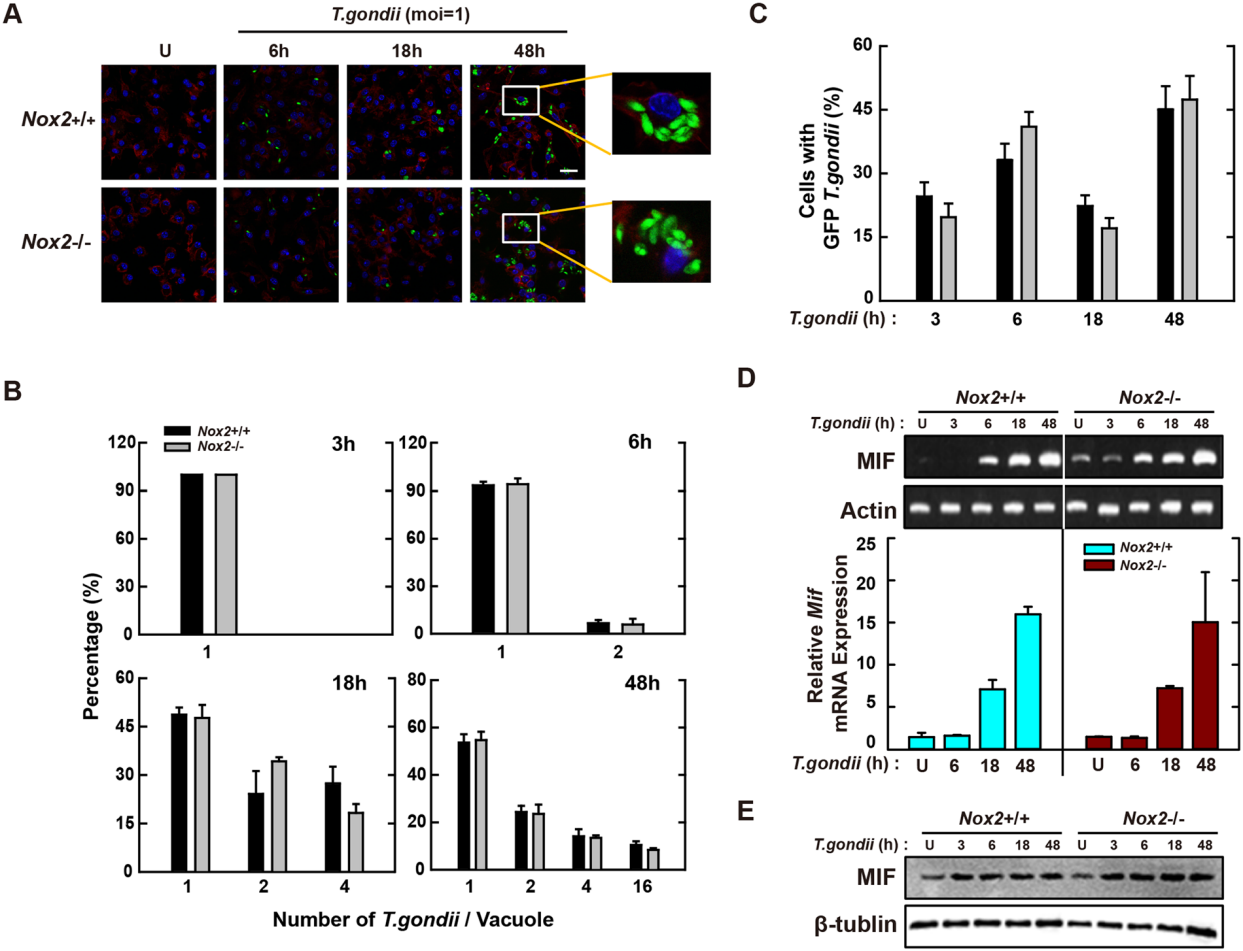
Macrophage-derived Nox2/gp91phox is not associated with induction of antiparasitic defense during *T*. *gondii* infection. (**A**–**C**) BMDMs from *Nox2* ^+/+^ and *Nox2* ^−/−^ mice were infected with GFP-RH strain (moi = 1) for the indicated time periods. (**A**) Cells were fixed and stained with Texas Red®-X phalloidin for labeling F-actin (red) for cytosolic fraction, and DAPI (blue) for nuclei and then analyzed for the number of GFP-RH strain using confocal microscopy (**B**,**C**) The number of GFP-RH strain per vacuole (for B) or of GFP-RH strain-infected cell (for C) were analyzed. Scale bar = 25 μm. Data are representative of five independent experiments (**D**,**E**) BMDMs from *Nox2* ^+/+^ and *Nox2* ^−/−^ mice were infected with *T*. *gondii* RH strain (moi = 1) for the indicated time periods. (**D**) The mRNA expression for *Mif* and *actb* was determined using semiquantitative RT-PCR (top) or qPCR (bottom) analysis. (**E**) Immunoblot analysis was performed to determine protein expression of MIF and β-tubulin. Data are representative of three independent experiments and are presented as means ± SD.

In phagocytes, Nox2/gp91phox is a major catalytic subunit of NADPH oxidase in generating ROS, which play an important role in host immune defenses against microbial pathogens^33^. Therefore, we assessed whether Nox2/gp91phox was necessary for the control of *T*. *gondii* infection and MIF expression in macrophages. We first investigated the effects of Nox2/gp91phox deficiency on intracellular survival of *T*. *gondii* in BMDMs. Confocal analysis showed that *T*. *gondii* proliferation was time-dependently increased in BMDMs; however, there was no obvious difference between *Nox2* ^+/+^ and *Nox2* ^−/−^ BMDMs in the number of tachyzoites per PV (Fig. 4A and B). Additionally, Nox2 deficiency did not altered the overall number of *T*. *gondii*-infected BMDMs (Fig. 4A and C).

Having found that ROS were involved in the activation of MIF expression in response to *T*. *gondii* infection, we next investigated whether MIF activation was regulated by the presence of the *Nox2* gene. Semi-quantitative RT-PCR (Fig. 4D, top) and real-time PCR analyses (Fig. 4D, bottom) indicated that *T*. *gondii* mediated the levels of MIF expression similarly in BMDMs with and without the *Nox2* gene. Moreover, protein levels of MIF were not affected by the absence of the *Nox2* gene in BMDMs (Fig. 4E). These data suggested that Nox2/gp91phox was not required for *T*. *gondii*-mediated MIF expression or its anti-parasitic activity in macrophages.

### The NADPH oxidase cytosolic subunit p47phox is not essential for the expression of MIF or controlling *T*. *gondii* infection in macrophages

**Figure 5:**
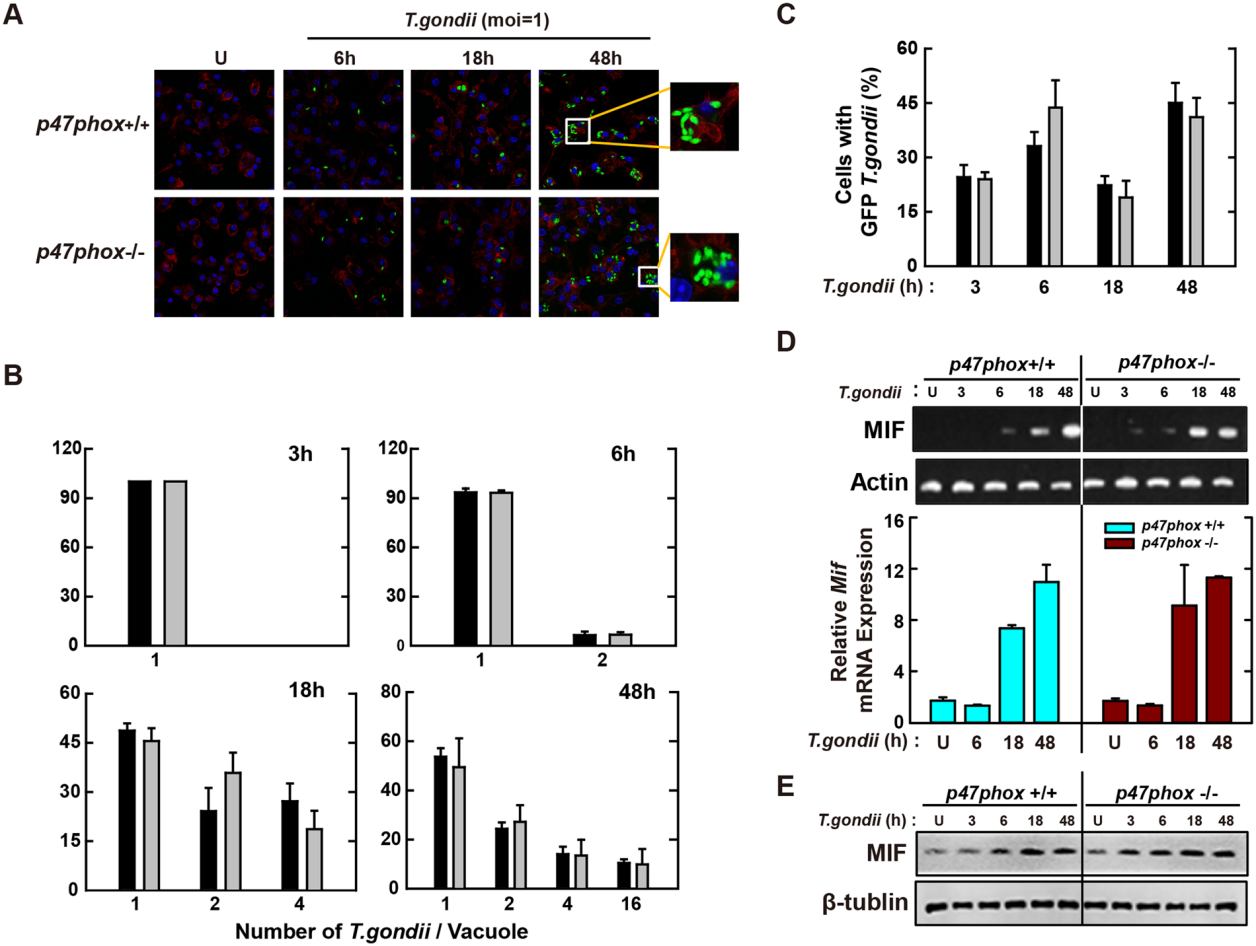
Macrophage-derived p47phox is not involved in antiparasitic defense during *T*. *gondii* infection. (**A**–**C**) BMDMs from *p47phox* ^+/+^ and *p47phox* ^−/−^ mice were infected with GFP-RH strain (moi = 1) for the indicated time periods. (**A**) Cells were fixed and stained with Texas Red®-X phalloidin for labeling F-actin (red) for cytosolic fraction, and DAPI (blue) for nuclei and then analyzed for the number of GFP-RH strain using confocal microscopy (**B**,**C**) The number of GFP-RH strain per vacuole (for B) or of GFP-RH strain-infected cell (for C) were analyzed. Scale bar = 25 μm. Data are representative of five independent experiments (**D**,**E**) BMDMs from *p47phox* ^+/+^ and *p47phox* ^−/−^ mice were infected with *T*. *gondii* RH strain (moi = 1) for the indicated time periods. (**D**) The mRNA expression for *Mif* and *actb* was determined using semiquantitative RT-PCR (top) or qPCR (bottom) analysis. (**E**) Immunoblot analysis was performed to determine protein expression of MIF and β-tubulin. Data are representative of three independent experiments and are presented as means ± SD.

Previous studies suggested that activated phagocytes produced ROS predominantly through the recruitment of NADPH oxidase family subunits to the plasma membrane^13, 33^. Among them, p47phox is a key regulatory cytosolic subunit of NADPH oxidase and genetic deficiency in human and rodent models has been associated with impaired clearance of diverse pathogens, including *Pseudomonas aeruginosa* ^34^, *Mycobacterium tuberculosis* ^35^, and *Propionibacterium acnes* ^36^. To evaluate whether p47phox was required for the intracellular control of *T*. *gondii* infection, primary BMDMs were isolated from *p47phox* ^+/+^ and *p47phox* ^−/−^ mice and then infected for various time periods with *T*. *gondii* (MOI = 1). As shown in Fig. 5A–C, the numbers of infected cells and the rates of intracellular proliferation of tachyzoites in PVs were similar between *p47phox* ^+/+^ and *p47phox* ^−/−^ BMDMs, suggesting that p47phox, like Nox2/gp91phox, was not required for parasite clearance by macrophages.

We also examined whether the absence of p47phox influenced the activation of MIF by infection with *T*. *gondii* in macrophages. As shown in Fig. 5D and E, mRNA (Fig. 5D) and protein levels (Fig. 5E) of MIF were upregulated effectively in *T*. *gondii*-treated wild-type (WT) macrophages in a time-dependent manner. However, there was no significant difference between *T*. *gondii*-infected *p47phox* ^+/+^ and *p47phox* ^−/−^ BMDMs in the levels of MIF mRNA (Fig. 5D) or protein (Fig. 5E). Taken together, these data indicated that p47phox was not essential for the activation of MIF expression or immune control of *T*. *gondii* infection in macrophages.

### Macrophage-derived Nox4 plays important roles in the intracellular clearance of *T*. *gondii* via modulating MIF expression

**Figure 6:**
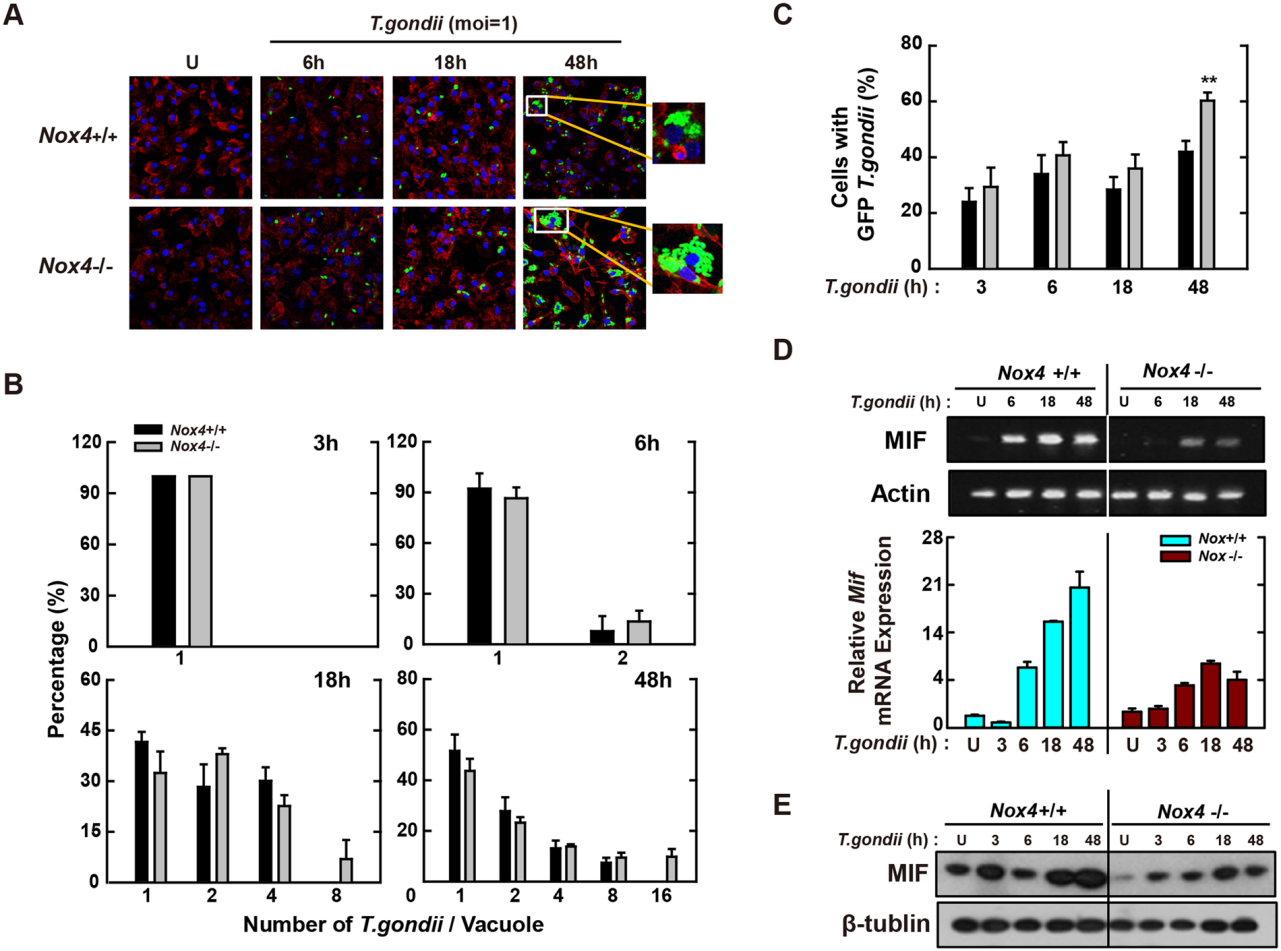
Nox4 is essential for the activation of protective immunity in *T*. *gondii*-infected macrophages. (**A**–**C**) BMDMs from *Nox4* ^+/+^ and *Nox4* ^−/−^ mice were infected with GFP-RH strain (moi = 1) for the indicated time periods. (**A**) Cells were fixed and stained with Texas Red®-X phalloidin for labeling F-actin (red) for cytosolic fraction, and DAPI (blue) for nuclei and then analyzed for the number of GFP-RH strain using confocal microscopy (**B**,**C**) The number of GFP-RH strain per vacuole (for B) or of GFP-RH strain-infected cell (for C) were analyzed. Scale bar = 25 μm. Data are representative of five independent experiments (**D**,**E**) BMDMs from *Nox4* ^+/+^ and *Nox4* ^−/−^ mice were infected with *T*. *gondii* RH strain (moi = 1) for the indicated time periods. (**D**) The mRNA expression for *Mif* and *actb* was determined using semiquantitative RT-PCR (top) or qPCR (bottom) analysis. (**E**) Immunoblot analysis was performed to determine protein expression of MIF and β-tubulin. Data are representative of three independent experiments and are presented as means ± SD. *P < 0.05, **P < 0.01, ***P < 0.001, two-tailed Student’s t-test.

Although initial studies suggested that Nox4 was unnecessary for the modulation of immune functions, it was reported recently that lipopolysaccharide (LPS)-induced ROS generation and NF-κB activation were mediated by a direct interaction between TLR4 and Nox4 in HEK293T cells^14^. Additionally, Nox4 was expressed constitutively and induced by stimulation with oxidized low-density lipoprotein (OxLDL) in human monocytes and macrophages^37^. Because we previously reported that host phosphoinositide 3-kinase (PI3K)/Akt-dependent Nox4 expression was essential for the control of *T*. *gondii* proliferation in the human retinal pigment epithelium cell line ARPE-19^38^, we further examined whether Nox4 was involved in the host immune defenses of macrophages against *T*. *gondii* infection. As above, we prepared primary BMDMs from *Nox4* ^+/+^ and *Nox4* ^−/−^ mice and then infected with them with *T*. *gondii* for the time periods indicated. As shown in Fig. 6A and B, the proliferation of intracellular parasite was enhanced in Nox4-deficient BMDMs. Moreover, Nox4 deficiency caused a significant increase in the number of infected cells (4-fold increase at 24 h; Fig. 6A and C).

We next assessed the MIF mRNA levels in *Nox4* ^+/+^ and *Nox4* ^−/−^ BMDMs using RT-PCR (Fig. 6D, top) and real-time PCR (Fig. 6D, bottom). Although MIF mRNA levels were increased from 6 h after infection with *T*. *gondii*, the increased expression levels of MIF mRNA were reduced significantly in *Nox4* ^−/−^ macrophages (Fig. 6D). These observations were also confirmed by the protein levels of MIF (Fig. 6E). Because the expression of various surface receptors related with host immune responses was impaired in *Mif* ^−/−^ macrophages infected with *T*. *gondii* ^24^, we assessed the effect of Nox4 deficiency on the mRNA levels of these receptors in *T*. *gondii*-infected macrophages. After infection with *T*. *gondii*, a lower *Ifngr1* mRNA (Fig. S5A) and a higher expression of *Tlr4* mRNA (Fig. S5C) were observed in *Nox4* ^−/−^ BMDMs. However, Nox4 deficiency did not affect the *Ccr5* mRNA level (Fig. S5B). Collectively, these findings suggest that Nox4 *is* involved in the *T*. *gondii*-mediated upregulation of MIF mRNA and protein levels, which is essential for the control of *T*. *gondii* proliferation and survival in macrophages.

### Nox4 is essential for resistance to *T*. *gondii* infection in mice

**Figure 7:**
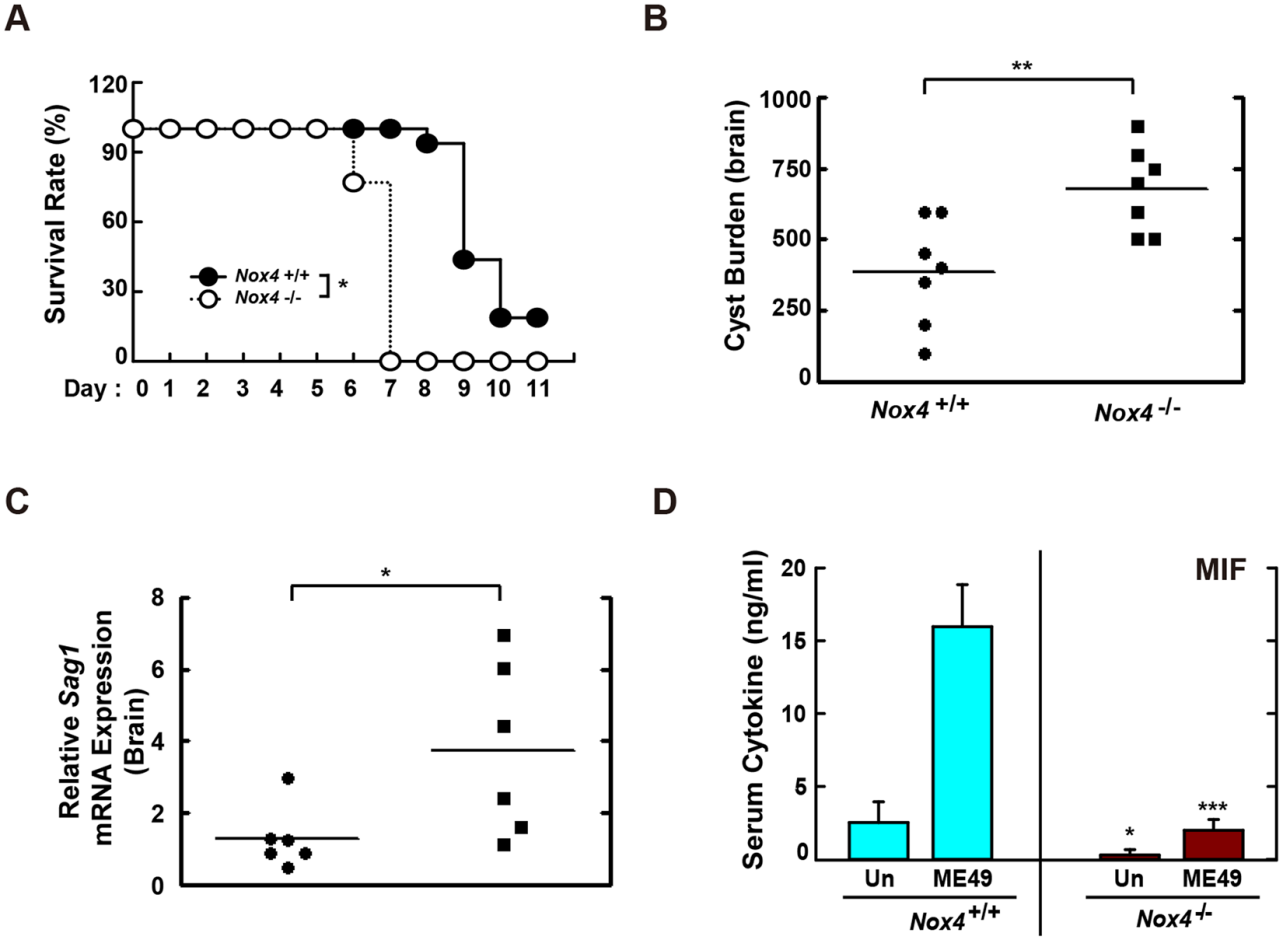
Nox4 is required for the host resistance against both RH and ME49 strain of *T*. *gondii*. (**A**) Survival of *Nox4* ^+/+^ and *Nox4* ^−/−^ mice (n = 13 per genotype) infected with 200 tachyzoites of *T*. *gondii* RH strain (i.p. injection). (**B**–**D**) *Nox4* ^+/+^ and *Nox4* ^−/−^ mice (n = 5 per genotype) were infected with 40 cysts of *T*. *gondii* ME49 strain (i.p. injection) for 20 days. (**B**) Number of cysts in brain of *Nox4* ^+/+^ and *Nox4* ^−/−^ mice were counted using a microscope. (**C**) The mRNA expression for *T*. *gondii*-specific gene *Sag1* in brain of *Nox4* ^+/+^ and *Nox4* ^−/−^ mice was evaluated by qPCR analysis. (**D**) Serum MIF levels from *Nox4* ^+/+^ and *Nox4* ^−/−^ mice were assessed by ELISA analysis. *P < 0.05, **P < 0.01, ***P < 0.001, compared with *Nox4* ^+/+^ mice infected with *T*. *gondii* (log-rank test (for A) or two-tailed Student’s t-test (for **B**–**D**).

In response to various infectious agents, intracellular ROS are generated and are involved in the activation of host immune responses^8, 39^. Moreover, Nox2-deficient mice showed impaired resistance to *Staphylococcus aureus* infection^40^. However, it has not been determined whether NADPH oxidase family proteins contribute to the protection of mice against *T*. *gondii* challenge. To assess the *in vivo* role(s) of Nox4 in *T*. *gondii* infection, *Nox4* ^+/+^ and *Nox4* ^−/−^ mice were challenged i.p. with the type I RH (200 parasites per mouse; Fig. 7A) or the type II ME49 strain (40 cysts per mouse; Fig. 7B–D). As shown in Fig. 7A, although *Nox4* ^+/+^ mice succumbed to the virulent RH strain from 9 d after i.p. injection, *Nox4* ^−/−^ mice were more susceptible to RH strain infection than *Nox4* ^+/+^ mice. We next examined the cyst burden in brain tissues at 20 d after challenge with the moderately virulent ME49 strain. The number of cysts was increased markedly in *Nox4* ^−/−^ mice versus *Nox4* ^+/+^ mice. Consistent with the increased number of cysts, the mRNA levels of *sag1*, a surface antigen of *T*. *gondii*, were also enhanced in the brains of infected *Nox4* ^−/−^ mice (Fig. 7C), which indicated that Nox4 is involved in controlling the conversion of tachyzoite/bradyzoite stage^41^.

Previous studies suggested that MIF participated in the host immune response to infection and a lack of MIF was associated with increased susceptibility in *T*. *gondii*-infected BALB/c or C57BL/6 mice^22, 24^. Thus, we next evaluated whether Nox4 deficiency affected the *T*. *gondii*-mediated generation of MIF protein in mice. When the mice were challenged with the ME49 strain, *Nox4* ^−/−^ mice had lower serum concentrations of MIF protein in both basal and *T*. *gondii*-infected conditions (Fig. 7D). In addition, the serum concentration of tumour necrosis factor alpha (TNF-α), but not that of interleukin (IL)-12p40, was significantly lower in *T*. *gondii*-infected *Nox4* ^−/−^ mice than in *T*. *gondii*-infected *Nox4* ^+/+^ mice (Fig. S6A,B). These results indicated that Nox4 was essential for host protection against *T*. *gondii* infection via MIF generation in mice.

## Discussion

MIF is a multifunctional cytokine and plays a key role in host antimicrobial defenses and the pathogenesis of inflammatory and autoimmune diseases, such as rheumatoid arthritis^42^, chronic colitis^43^, and severe sepsis^44, 45^. Indeed, previous studies have shown that MIF is closely associated with host resistance or susceptibility to diverse protozoan infections. Flores *et al*. found that C57BL/6 and BALB/c MIF KO mice were more susceptible to both infection with the virulent RH and the avirulent ME49 strain because of impaired production of proinflammatory cytokines and significant increases in infected peritoneal macrophages, cyst burden in the brain, and liver damage than WT mice^24^. Here we have shown that the survival of *Nox4* ^−/−^ mice infected with RH strain were significantly lower than that of *Nox4* ^+/+^ mice (Fig. 7A). Moreover, the serum concentrations of TNF-α and MIF, but not that of IL-12p40, were lower in ME49-infected *Nox4* ^*−/−*^ mice than in ME49-infected *Nox4* ^+/+^ mice (Figs 7D and S6A,B). This is in part consistent with previous reports showing that increased susceptibility to *T*. *gondii* infection is closely associated with impaired production of inflammatory cytokines.

Treatment with recombinant MIF (rMIF) in the first (25 ng/mL) and third (100 ng/mL) trimester resulted in significant decreases in the parasitic burden^46^. In addition, administration of rMIF into *T*. *gondii*-infected MIF-deficient mice significantly improved the survival, as well as WT mice^47^. Because of this possibility, many investigators have sought to evaluate the clinical possibility of MIF recombinant protein and MIF antagonism as a therapeutic target for parasitic diseases^15^. In the present study, we also found that the mRNA and protein levels of MIF were upregulated significantly upon *T*. *gondii* RH strain infection in primary macrophages, in a time- and MOI-dependent manner (Fig. 1A–C). Moreover, *Nox4* ^+/+^ mice infected with the ME49 strain showed markedly increased levels of MIF in serum (Fig. 7D). Knockdown of Mif with a letiviral vector containing shMIF enhanced the survival of *T*. *gondii* in primary macrophages (Fig. 1B,C and Fig. S2). This demonstrates that MIF is involved in control of *T*. *gondii* infection in macrophages.

Although MIF generation upon *T*. *gondii* infection is crucial for the anti-toxoplasma effects, however, the precise molecular mechanism(s) mediating *T*. *gondii*-induced upregulation of MIF generation still have not been investigated. We found that MIF production was attenuated significantly in the serum of Nox4-deficient mice infected with the ME49 strain (Fig. 7D). Similarly, primary macrophages isolated from Nox4-deficient mice showed marked decreases in MIF mRNA (Fig. 6D) and protein (Fig. 6E) levels, whereas the genetic deletion of Nox2 (Fig. 4D and E) or p47phox (Fig. 5D and E) had no such effect. A recent study using the human retinal pigment epithelium cell line ARPE-19 suggested that *T*. *gondii* infection or excretory/secretory protein (ESP) treatment resulted in the suppression of Nox4 gene expression and, consequently, inhibition of H_2_O_2_-induced intracellular ROS generation through the PI3K/Akt signaling pathway, which is critical for *T*. *gondii* survival and proliferation^38^. Based on these results, we hypothesized that Nox4-derived ROS generation played a role in the activation of MIF gene expression.

In phagocytes, ROS are generally produced through a specific process with the NADPH oxidase enzyme or a non-specific process with the mitochondrial respiratory chain; they act as effector molecules having various biosynthetic roles and acting in host innate defenses for killing microbes^13, 48, 49^. Although gp91phox/Nox2 is an important component for generating ROS in phagocytic cells, other Nox isoforms, such as Nox1 and 4 and Duox1 and 2, are also expressed in rat hepatocytes^50^. Moon *et al*. found that Nox4 deficiency resulted in the suppression of carnitine palmitoyltransferase 1 A and the NACHT, LRR and PYD domains-containing protein 3 (NLRP3) inflammasome, and reported improved survival in Nox4-deficient mice infected with *Streptococcus pneumoniae* ^51^. Furthermore, LPS stimulation resulted in the activation of ROS generation and NF-κB signaling by a direct interaction of TLR4 with Nox4 in HEK293T cells^14^. In the present study, we found that Nox4-, but not Nox2- or p47phox-, deficient mice succumbed to infection with the highly virulent type I RH strain faster than WT mice and also failed to control the growth of the moderately virulent ME49 strain in the brain (Fig. 7A–C). These findings suggest that Nox4 is essential for the activation of host protection against *T*. *gondii* infection, which may be related to MIF production.

To date, many studies support that ROS are essential as signaling molecules in regulating a broad range of physiological and pathological responses, including inflammation, host immune response, and cell death^52, 53^. Matsuzawa *et al*. found that the ROS-dependent TNF receptor associated factor (TRAF6)-apoptosis signal-regulating kinase 1 (ASK1)-p38 pathway was important in the TLR4-mediated activation of innate immune responses^54^. Moreover, our previous results demonstrated that TLR-mediated ROS generation was required for the production of proinflammatory cytokines via the TRAF6-ASK1-MAPK axis following the control of intracellular mycobacterial survival^28, 35^. Additionally, the oxidative burst after LPS stimulation contributed to the production of interleukin (IL)-8 through the activation of NF-κB signaling in the human monocyte/macrophage cell line THP-1^55^. In this study, we extended those findings by demonstrating that *T*. *gondii*-induced intracellular ROS generation was required for the activation of MAPK (Fig. 2D) and NF-κB (Fig. 3D) signaling. Moreover, pharmacological inhibition of MAPK (Fig. 2B) and NF-κB (Fig. 3C) signaling markedly attenuated *T*. *gondii*-induced MIF mRNA and protein levels in BMDMs.

Taken together, our findings demonstrated a mechanistic division between *T*. *gondii*-induced intracellular ROS generation and MIF activation, which may contribute to protective immunity against *T*. *gondii* infection. Additionally, our results suggested that macrophage-derived Nox4, but not Nox2 or p47phox, acts as an essential enzyme in intracellular ROS generation and provides an intracellular signaling pathway(s), leading to *T*. *gondii*-mediated induction of MIF expression *in vitro* in primary macrophages. Our results provide new insights into the role of ROS signaling in *T*. *gondii* infection.

## Electronic supplementary material

Supplementary information
