# Supplementary material for: NADPH oxidase 4 is required for the generation of macrophage migration inhibitory factor and host defense against Toxoplasma gondii infection
Source: Sci Rep. 2017 Jul 25;7:6361. doi: 10.1038/s41598-017-06610-4 (PMC5526938; doi:10.1038/s41598-017-06610-4)

NADPH oxidase 4 is required for the generation of macrophage migration inhibitory factor and host defense against *Toxoplasma gondii* infection.

Ji Hye Kim[1,2¶], Jina Lee[1,2¶], Su-Jin Bae[1,2], Ye-Eun Kim[1], Byung-Joon Park[1,2], Jae-Won Choi[1,2], Jaeyul Kwon[2,3], Guang-Ho Cha[1,2], Heon Jong Yoo[4], Eun-Kyeong Jo[2,5], Yun Soo Bae[6], Young-Ha Lee[1,2*], Jae-Min Yuk[1,2*]

Supplementary Information

Supplementary Figure S1. *T.gondii* infection activates the mRNA expression of *Mif* in BMDMs.

(A) BMDMs were infected with *T.gondii* RH strain (moi = 1) for the indicated time periods and then cell lysate was collected. The mRNA expression for *Mif* and *actb* was determined using semiquantitative RT-PCR (top) or quantitative real-time PCR analysis (qPCR; bottom) analysis. (B and C) BMDMs were infected with *T.gondii* RH strain (moi = 0.2, 0.5, and 1) for 18 h. (B) Cell lysates were subjected to SDS-PAGE, followed by western blot analysis using anti-MIF and anti-β-tubulin Abs. (C) The mRNA expression for *Mif* and *actb* was determined using semiquantitative RT-PCR (top) or qPCR (bottom) analysis. Data are representative of three independent experiments and are presented as means ± SD. ***$P < 0.001$, two-tailed Student's t-test.

Supplementary Figure S2. Knockdown of MIF in macrophages leads to an increased expression of *sag1* mRNA.

BMDM were transduced with lentiviruses expressing shNS or shMIF (at MOI of 1, 5, and 20 for top or 5 for bottom) for 48 h with polybrene (8 μg/mL) and then infected with *T.gondii* RH strain for 18 h. The mRNA expression for *Mif* and *actb* was determined using semiquantitative RT-PCR (top). qPCR were assessed to determine *sag1* mRNA expression in whole-cell lysates (bottom).

Supplementary Figure S3. *T.gondii* infection induces the generation of hydrogen peroxides in BMDMs.

BMDMs were infected with *T.gondii* RH strain (moi=1) for indicated times and then stained with

H2DCFDA (20 μM) for 20 min. Intracellular ROS generation was measured using a fluorescence microscope. Scale bar = 100 μm. H_2O_2 (1 mM, 30 min) was used for positive control.

Supplementary Figure S4. *T.gondii* infection enhances NF-κB transcriptional activity in Raw 264.7 cells in a time - or moi - dependent manner.

(A and B) Raw 264.7 cells were transfected with plasmids carrying NF-κB luciferase reporter constructs before *T.gondii* RH strain (moi = 1, for A; indicated moi, for B) for various time periods (for A) or 18 h (for B). Luciferase assays were performed based on normalization to the β-galactosidase activity. Data are representative of three independent experiments and are presented as means ± SD. *P < 0.05, **P < 0.01, ***P < 0.001, two-tailed Student's t-test.

Supplementary Figure S5. Analysis of the mRNA expression of various surface receptors on *T.gondii*-infected *Nox4*$^{+/+}$ and *Nox4*$^{-/-}$ BMDMs.

(A-C) BMDMs from *Nox4*$^{+/+}$ and *Nox4*$^{-/-}$ mice were infected with *T.gondii* RH strain (moi = 1) for the indicated time periods and then cell lysate was collected. The mRNA expression for *Ifngr1* (for A), *Ccr5* (for B), and *Tlr4* (for C) were determined using qPCR analysis. Data are representative of three independent experiments and are presented as means ± SD. *P < 0.05, ***P < 0.001, two-tailed Student's t-test.

Supplementary Figure S6. Nox4-deficient mice exhibit an impaired production of inflammatory cytokine TNFα in response to *T.gondii* ME49 strain infection.

(A and B) *Nox4*$^{+/+}$ and *Nox4*$^{-/-}$ mice (n = 5 per genotype) were infected with 40 cysts of *T.gondii*

ME49 strain (i.p. injection) for 20 days. Levels of Serum TNFα (for A) and IL-12p40 (for B) from *Nox4*$^{+/+}$ and *Nox4* $^{-/-}$ mice were assessed by ELISA analysis. ***P < 0.001, compared with *Nox4*$^{+/+}$ mice infected with *T.gondii* (two-tailed Student's t-test).

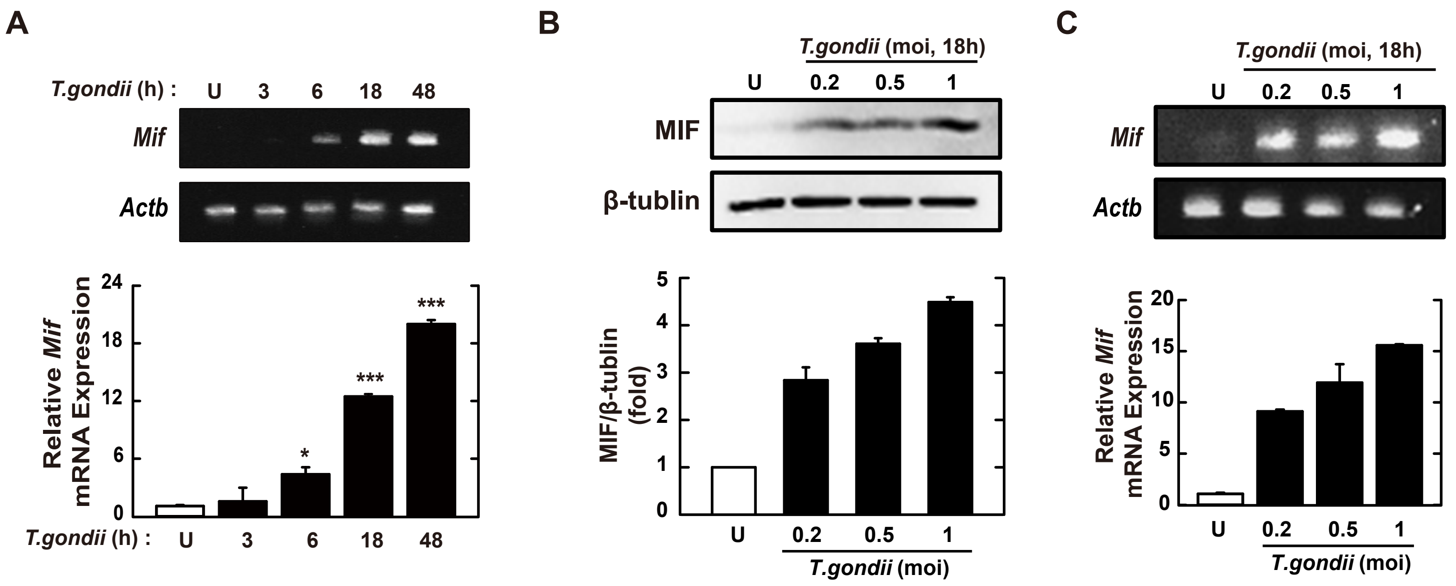
A
T.gondii (h) :
U
3
6
18
48
Mif
Actb
Relative Mif mRNA Expression
24
18
12
6
0
*
***
***
B
T.gondii (moi, 18h)
0.2
0.5
1
MIF
β-tublin
MIF/β-tublin (fold)
5
4
3
2
1
0
T.gondii (moi)
C
Relative Mif mRNA Expression
20
15
10
5
0

Supplementary Figure S2

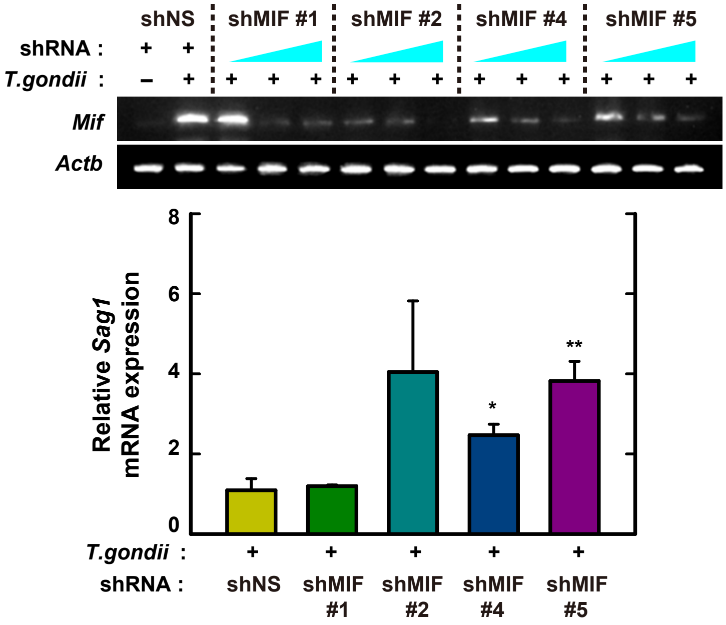

Supplementary Figure S3

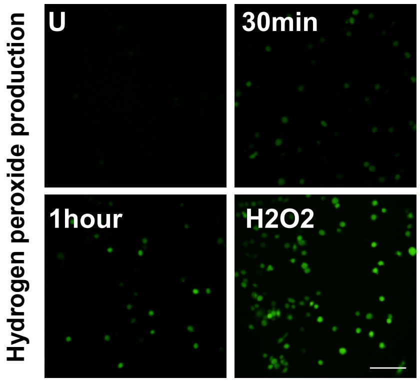

Supplementary Figure S4

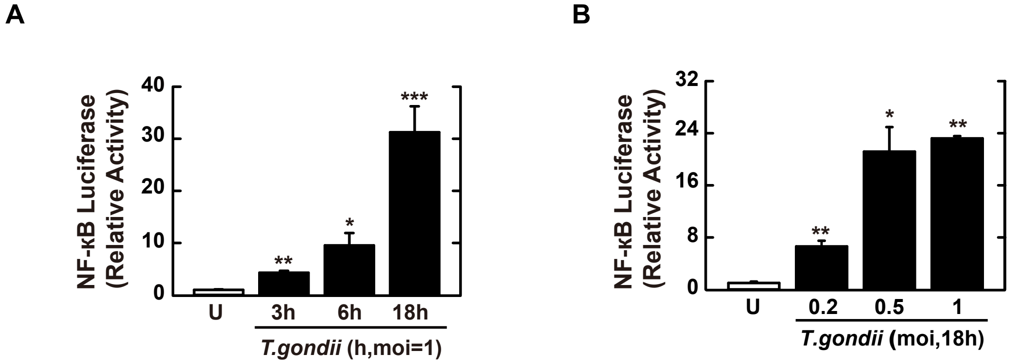

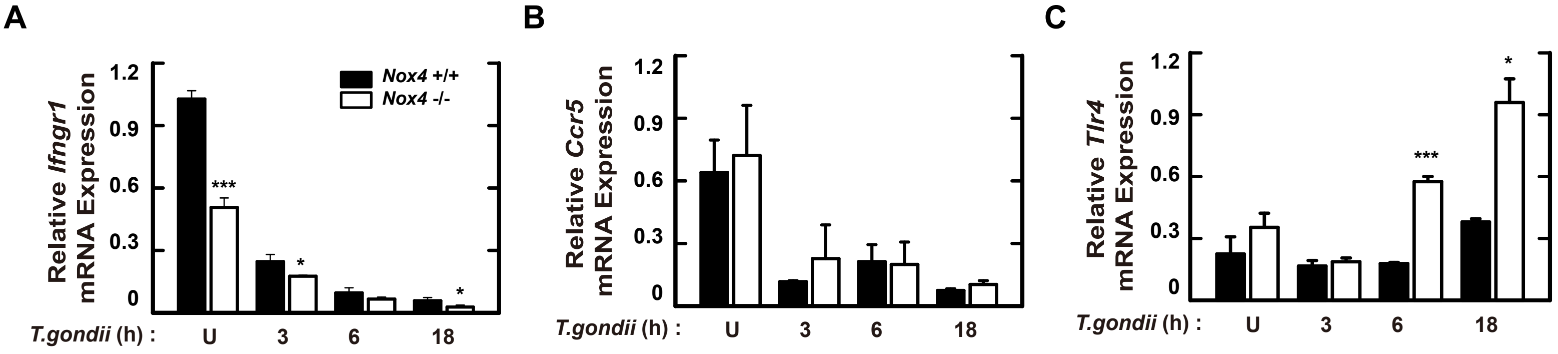
A
Relative Ifngr1 mRNA Expression
1.2
0.9
0.6
0.3
0
Nox4 +/+
Nox4 -/-
***
*
*
T.gondii (h) :
U
3
6
18
B
Relative Ccr5 mRNA Expression
1.2
0.9
0.6
0.3
0
T.gondii (h) :
U
3
6
18
C
Relative Tlr4 mRNA Expression
1.2
0.9
0.6
0.3
0
***
*
T.gondii (h) :
U
3
6
18

Supplementary Figure S6

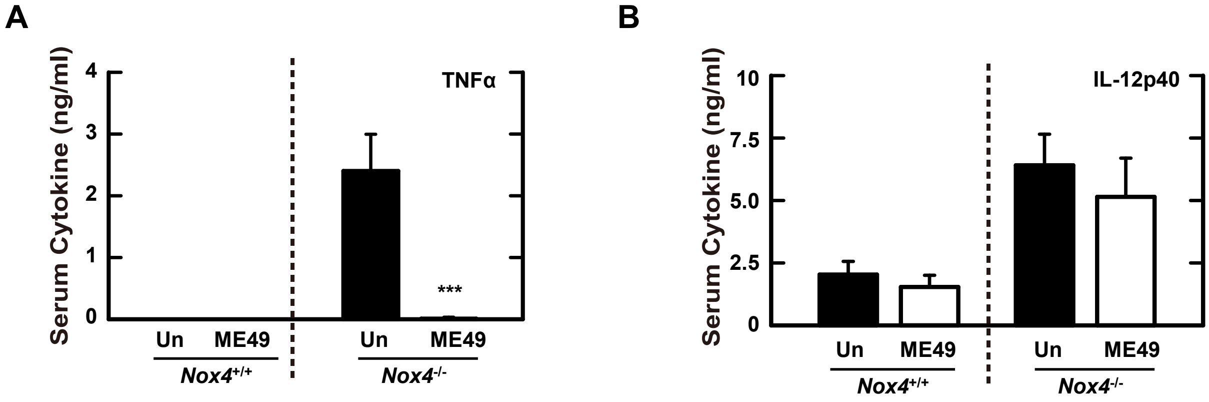

Supplement: Supplementary file 1 — Supplementary information [file 41598_2017_6610_MOESM1_ESM.pdf]
